# Hexaaqua­magnesium(II) bis­(pyridinium-2,6-dicarboxyl­ate)

**DOI:** 10.1107/S1600536810046696

**Published:** 2010-11-17

**Authors:** Hoda Pasdar, Sanaz Heidari, Hossein Aghabozorg, Behrouz Notash

**Affiliations:** a Department of Chemistry, Islamic Azad University, North Tehran Branch, Tehran, Iran; b Department of Chemistry, Shahid Beheshti University, G. C., Evin, Tehran 1983963113, Iran

## Abstract

In the title compound, [Mg(H_2_O)_6_](C_7_H_4_NO_4_)_2_, a single six-coordinate Mg^2+^ cation (site symmetry 2/*m*) is bonded to six water mol­ecules in a distorted octa­hedral geometry. The crystal packing between the complex cation and the zwitterionic organic cation (*m* symmetry) is stabilized by inter­molecular O—H⋯O hydrogen bonds and weak inter­molecular C—H⋯O inter­actions.

## Related literature

For background to proton-transfer compounds, see: Aghabozorg *et al.* (2008 ▶). For related structures, see: Aghabozorg *et al.* (2005 ▶); Grossel *et al.* (2006 ▶); Ptasiewicz-Bak & Leciejewicz (2003 ▶); Dale *et al.* (2003 ▶); Yang *et al.* (2005 ▶); Kariuki & Jones (1989 ▶)
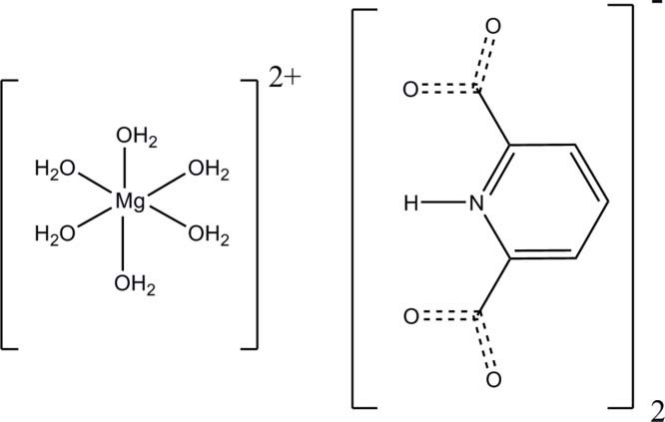


## Experimental

### 

#### Crystal data

[Mg(H_2_O)_6_](C_7_H_4_NO_4_)_2_
*M* *_r_* = 464.63
Monoclinic, 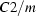

*a* = 13.432 (3) Å
*b* = 11.108 (2) Å
*c* = 6.5845 (13) Å
β = 92.79 (3)°
*V* = 981.3 (3) Å^3^
*Z* = 2
Mo *K*α radiation
μ = 0.17 mm^−1^
*T* = 298 K
0.35 × 0.30 × 0.15 mm

#### Data collection

Stoe IPDS II diffractometer
Absorption correction: numerical (*X-RED* and *X-SHAPE*; Stoe & Cie, 2005 ▶) *T*_min_ = 0.940, *T*_max_ = 0.973
5499 measured reflections
1383 independent reflections
1178 reflections with *I* > 2σ(*I*)
*R*_int_ = 0.031

#### Refinement

*R*[*F* ^2^ > 2σ(*F* ^2^)] = 0.038
*wR*(*F* ^2^) = 0.095
*S* = 1.12
1383 reflections
94 parameters
H atoms treated by a mixture of independent and constrained refinement
Δρ_max_ = 0.33 e Å^−3^
Δρ_min_ = −0.18 e Å^−3^

### 

Data collection: *X-AREA* (Stoe & Cie, 2005 ▶); cell refinement: *X-AREA*; data reduction: *X-AREA*; program(s) used to solve structure: *SHELXS97* (Sheldrick, 2008 ▶); program(s) used to refine structure: *SHELXL97* (Sheldrick, 2008 ▶); molecular graphics: *ORTEP-3 for Windows* (Farrugia, 1997 ▶); software used to prepare material for publication: *WinGX* (Farrugia, 1999 ▶).

## Supplementary Material

Crystal structure: contains datablocks I, glolbal. DOI: 10.1107/S1600536810046696/jj2069sup1.cif

Structure factors: contains datablocks I. DOI: 10.1107/S1600536810046696/jj2069Isup2.hkl

Additional supplementary materials:  crystallographic information; 3D view; checkCIF report

## Figures and Tables

**Table 1:** Hydrogen-bond geometry (Å, °)

*D*—H⋯*A*	*D*—H	H⋯*A*	*D*⋯*A*	*D*—H⋯*A*
O3—H3⋯O1	0.862 (19)	1.834 (19)	2.6940 (14)	174.6 (19)
O4—H4⋯O2	0.85 (2)	1.93 (2)	2.7758 (14)	171 (2)
O5—H5⋯O2^i^	0.89 (2)	1.92 (2)	2.7960 (14)	167.5 (19)
C1—H1⋯O5^ii^	0.93	2.58	3.308 (3)	136

Symmetry codes: (i) 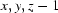; (ii) 
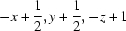
.

**Fig. 1.**
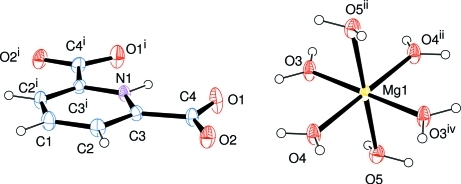
The molecular structure of [Mg(H2O)6][pyH-2,6-dc]2 with displacement ellipsoids drawn at 30% probability level. Symmetry codes: (i: x, 1 - y, z; ii: 1 - x, y, -z; iv: 1 - x, -y, -z).

**Fig. 2.**
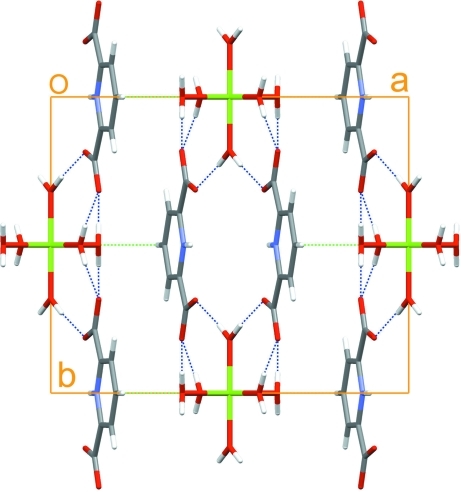
The packing diagram of [Mg(H2O)6][pyH-2,6-dc]2 viewed down the c-axis. The intra and intermolecular O—H···O and intermolecular C—H···O hydrogen bonds are shown as blue and green dashed lines, respectively.

## supplementary crystallographic information

### Comment

Pyridine-2,6-dicarboxylic acid is commonly used as proton donor in proton transfer systems (Aghabozorg *et al.* 2008). It has been reported that the carboxylate groups are deprotonated and the pyridine ring is protonated in compounds containing pyridine-2,6-dicarboxylic acid( Aghabozorg *et al.* 2005; Grossel *et al.* 2006).  In addition, the formation of a six-coordinated magnesium (II) ion by water molecules in aqueous solution in the presence of poly carboxylic acids has been observed (Dale *et al.* 2003; Ptasiewicz-Bak & Leciejewicz 2003; Yang *et al.* 2005).  The structure of hexa-aquamagnesium(II) pyrazine-2,6-dicarboxylate, [Mg(H_2_O)_6_][pz-2,6-dc], has also been reported which exhibits hydrogen bonding between the cationic magnesium species and a pyrazine-2,6-dicarboxylate anion (Ptasiewicz-Bak & Leciejewicz 2003).

In the title compound, [Mg(H_2_O)_6_][pyH-2,6-dc]_2_, the cation is comprised of a six-coordinate Mg^II^ ion bound by water molecules in a distorted octahedral geometry.  The dianion is comprised of a pyridine-2,6-dicarboxylic acid group (Fig. 1).  Bond lengths and angles for Mg—O are in normal ranges. Crystal packing is stabilized by O—H···O intra and intermolecular hydrogen bonds and weak C—H···O intermolecular hydrogen bond interactions with the coordinated water molecules (Fig. 2). The pyridine ring in the dianion is protonated and the two carboxylic acid groups are deprotonated forming a proton transfer fragment.

### Experimental

A solution of pyridine-2,6-dicarboxylic acid (pydcH_2_) (0.1671 g, 1 mmol) in ethanol (20 ml) was added to a solution of pyridazine (pydz) (0.072 ml, 1 mmol) in ethanol (8 ml) and stirred for 2 hrs. Then an aqueous solution of Mg(NO_3_)_2_.6H_2_O (0.1282 g, 0.5 mmol) was added to mixture of pydcH_2_-pydz and stirred for 1 h. 1 mL DMSO was then added to the mixture to clear the solution and stirred for more 2 hrs. Slow evaporation of the resulting solution gave colorless crystals of the title compound after three weeks which were suitable for X-ray analysis (decomposition > 260 °C).

### Refinement

The hydrogen atoms from the water molecules and pyridinium group were found in a difference Fourier map and refined isotropically without restraint. The C—H protons of the aromatic ring were positioned geometrically and refined as riding atoms, with C–H = 0.93Å and *U*_iso_(H) = 1.2*U*_eq_(C).

### Crystal data

**Table:**

[Mg(H_2_O)_6_](C_7_H_4_NO_4_)_2_	*F*(000) = 484.0
*M**_r_* = 464.63	*D*_x_ = 1.572 Mg m^−^^3^
Monoclinic, *C*2/*m*	Mo *K*α radiation, λ = 0.71073 Å
Hall symbol: -C 2y	Cell parameters from 1383 reflections
*a* = 13.432 (3) Å	θ = 2.4–29.1°
*b* = 11.108 (2) Å	µ = 0.17 mm^−^^1^
*c* = 6.5845 (13) Å	*T* = 298 K
β = 92.79 (3)°	Plate, colorless
*V* = 981.3 (3) Å^3^	0.35 × 0.30 × 0.15 mm
*Z* = 2	

### Data collection

**Table:**

Stoe IPDS II diffractometer	1383 independent reflections
Radiation source: fine-focus sealed tube	1178 reflections with *I* > 2σ(*I*)
graphite	*R*_int_ = 0.031
Detector resolution: 0.15 pixels mm^-1^	θ_max_ = 29.1°, θ_min_ = 2.4°
rotation method scans	*h* = −17→18
Absorption correction: numerical (*X-RED* and *X-SHAPE*; Stoe & Cie, 2005)	*k* = −14→15
*T*_min_ = 0.940, *T*_max_ = 0.973	*l* = −9→8
5499 measured reflections	

### Refinement

**Table:**

Refinement on *F*^2^	Primary atom site location: structure-invariant direct methods
Least-squares matrix: full	Secondary atom site location: difference Fourier map
*R*[*F*^2^ > 2σ(*F*^2^)] = 0.038	Hydrogen site location: inferred from neighbouring sites
*wR*(*F*^2^) = 0.095	H atoms treated by a mixture of independent and constrained refinement
*S* = 1.12	*w* = 1/[σ^2^(*F*_o_^2^) + (0.0414*P*)^2^ + 0.5177*P*] where *P* = (*F*_o_^2^ + 2*F*_c_^2^)/3
1383 reflections	(Δ/σ)_max_ = 0.001
94 parameters	Δρ_max_ = 0.33 e Å^−^^3^
0 restraints	Δρ_min_ = −0.18 e Å^−^^3^

### Special details

**Table:**

Experimental. shape of crystal determined optically
Geometry. All e.s.d.'s (except the e.s.d. in the dihedral angle between two l.s. planes) are estimated using the full covariance matrix. The cell e.s.d.'s are taken into account individually in the estimation of e.s.d.'s in distances, angles and torsion angles; correlations between e.s.d.'s in cell parameters are only used when they are defined by crystal symmetry. An approximate (isotropic) treatment of cell e.s.d.'s is used for estimating e.s.d.'s involving l.s. planes.
Refinement. Refinement of *F*^2^ against ALL reflections. The weighted *R*-factor *wR* and goodness of fit *S* are based on *F*^2^, conventional *R*-factors *R* are based on *F*, with *F* set to zero for negative *F*^2^. The threshold expression of *F*^2^ > σ(*F*^2^) is used only for calculating *R*-factors(gt) *etc*. and is not relevant to the choice of reflections for refinement. *R*-factors based on *F*^2^ are statistically about twice as large as those based on *F*, and *R*- factors based on ALL data will be even larger.

### Fractional atomic coordinates and isotropic or equivalent isotropic displacement parameters (Å^2^)

**Table:**

	*x*	*y*	*z*	*U*_iso_*/*U*_eq_	
Mg1	0.5000	0.0000	0.0000	0.0249 (2)	
O1	0.40364 (11)	0.31353 (9)	0.27234 (16)	0.0499 (3)	
O2	0.36594 (9)	0.18375 (8)	0.51657 (15)	0.0396 (3)	
O3	0.5000	0.18182 (12)	0.0000	0.0360 (3)	
O4	0.41819 (12)	0.0000	0.2566 (2)	0.0357 (3)	
O5	0.36739 (11)	0.0000	−0.1931 (2)	0.0341 (3)	
N1	0.37142 (12)	0.5000	0.4931 (2)	0.0257 (3)	
C1	0.31770 (16)	0.5000	0.8796 (3)	0.0347 (4)	
H1	0.2989	0.5000	1.0137	0.042*	
C2	0.33156 (11)	0.39121 (12)	0.78070 (19)	0.0312 (3)	
H2	0.3225	0.3186	0.8476	0.037*	
C3	0.35898 (9)	0.39277 (10)	0.58164 (18)	0.0253 (3)	
C4	0.37779 (11)	0.28571 (11)	0.4447 (2)	0.0310 (3)	
H3	0.4708 (14)	0.2281 (19)	0.084 (3)	0.053 (5)*	
H4	0.4046 (15)	0.0617 (19)	0.327 (3)	0.057 (6)*	
H5	0.3664 (16)	0.0661 (19)	−0.270 (3)	0.065 (6)*	
H1A	0.3899 (19)	0.5000	0.366 (4)	0.046 (7)*	

### Atomic displacement parameters (Å^2^)

**Table:**

	*U*^11^	*U*^22^	*U*^33^	*U*^12^	*U*^13^	*U*^23^
Mg1	0.0367 (5)	0.0173 (4)	0.0211 (4)	0.000	0.0062 (3)	0.000
O1	0.0892 (9)	0.0278 (5)	0.0351 (5)	0.0102 (5)	0.0267 (6)	−0.0011 (4)
O2	0.0626 (7)	0.0192 (4)	0.0374 (5)	0.0041 (4)	0.0054 (5)	0.0011 (4)
O3	0.0602 (10)	0.0177 (6)	0.0315 (7)	0.000	0.0172 (6)	0.000
O4	0.0566 (9)	0.0226 (6)	0.0296 (7)	0.000	0.0179 (6)	0.000
O5	0.0467 (8)	0.0295 (7)	0.0262 (6)	0.000	0.0034 (6)	0.000
N1	0.0353 (8)	0.0203 (7)	0.0220 (6)	0.000	0.0081 (6)	0.000
C1	0.0486 (12)	0.0344 (10)	0.0217 (8)	0.000	0.0080 (7)	0.000
C2	0.0422 (8)	0.0253 (6)	0.0265 (6)	0.0000 (5)	0.0059 (5)	0.0048 (5)
C3	0.0303 (6)	0.0192 (5)	0.0267 (5)	0.0018 (4)	0.0036 (4)	0.0007 (4)
C4	0.0410 (7)	0.0210 (6)	0.0312 (6)	0.0050 (5)	0.0048 (5)	−0.0027 (5)

### Geometric parameters (Å, °)

**Table:**

Mg1—O3^i^	2.0197 (14)	O5—H5	0.89 (2)
Mg1—O3	2.0197 (14)	N1—C3^ii^	1.3401 (13)
Mg1—O4^i^	2.0601 (15)	N1—C3	1.3401 (13)
Mg1—O4	2.0601 (15)	N1—H1A	0.89 (3)
Mg1—O5^i^	2.1375 (16)	C1—C2	1.3897 (16)
Mg1—O5	2.1375 (16)	C1—C2^ii^	1.3897 (16)
O1—C4	1.2422 (17)	C1—H1	0.9300
O2—C4	1.2406 (16)	C2—C3	1.3788 (17)
O3—H3	0.862 (19)	C2—H2	0.9300
O4—H4	0.85 (2)	C3—C4	1.5208 (17)
			
O3^i^—Mg1—O3	180.0	Mg1—O5—H5	109.1 (13)
O3^i^—Mg1—O4^i^	90.0	C3^ii^—N1—C3	125.46 (15)
O3—Mg1—O4^i^	90.0	C3^ii^—N1—H1A	117.27 (8)
O3^i^—Mg1—O4	90.0	C3—N1—H1A	117.27 (8)
O3—Mg1—O4	90.0	C2—C1—C2^ii^	120.82 (17)
O4^i^—Mg1—O4	180.00 (8)	C2—C1—H1	119.6
O3^i^—Mg1—O5^i^	90.0	C2^ii^—C1—H1	119.6
O3—Mg1—O5^i^	90.0	C3—C2—C1	118.87 (12)
O4^i^—Mg1—O5^i^	91.46 (6)	C3—C2—H2	120.6
O4—Mg1—O5^i^	88.54 (6)	C1—C2—H2	120.6
O3^i^—Mg1—O5	90.0	N1—C3—C2	117.99 (12)
O3—Mg1—O5	90.0	N1—C3—C4	114.17 (11)
O4^i^—Mg1—O5	88.54 (6)	C2—C3—C4	127.84 (11)
O4—Mg1—O5	91.46 (6)	O2—C4—O1	128.49 (12)
O5^i^—Mg1—O5	180.00 (8)	O2—C4—C3	117.37 (12)
Mg1—O3—H3	126.6 (13)	O1—C4—C3	114.14 (11)
Mg1—O4—H4	125.5 (14)		
			
C2^ii^—C1—C2—C3	−0.3 (3)	N1—C3—C4—O2	−178.85 (14)
C3^ii^—N1—C3—C2	−0.3 (3)	C2—C3—C4—O2	1.3 (2)
C3^ii^—N1—C3—C4	179.77 (12)	N1—C3—C4—O1	1.23 (19)
C1—C2—C3—N1	0.3 (2)	C2—C3—C4—O1	−178.66 (14)
C1—C2—C3—C4	−179.83 (15)		

Symmetry codes: (i) −*x*+1, −*y*, −*z*; (ii) *x*, −*y*+1, *z*.

### Hydrogen-bond geometry (Å, °)

**Table:**

*D*—H···*A*	*D*—H	H···*A*	*D*···*A*	*D*—H···*A*
O3—H3···O1	0.862 (19)	1.834 (19)	2.6940 (14)	174.6 (19)
O4—H4···O2	0.85 (2)	1.93 (2)	2.7758 (14)	171 (2)
O5—H5···O2^iii^	0.89 (2)	1.92 (2)	2.7960 (14)	167.5 (19)
C1—H1···O5^iv^	0.93	2.58	3.308 (3)	136

Symmetry codes: (iii) *x*, *y*, *z*−1; (iv) −*x*+1/2, *y*+1/2, −*z*+1.
